# Financial Performance Gaps Between Critical Access Hospitals and Other Acute Care Hospitals

**DOI:** 10.1001/jamahealthforum.2024.3959

**Published:** 2024-12-20

**Authors:** Christopher Whaley, Marilyn Bartlett, Ge Bai

**Affiliations:** 1 Department of Health Services Policy and Practice, Brown University School of Public Health, Providence, Rhode Island; 2 National Academy for State Health Policy, Washington, DC; 3 Carey Business School, Johns Hopkins University, Baltimore, Maryland; 4 Bloomberg School of Public Health, Johns Hopkins University, Baltimore, Maryland

## Abstract

This cross-sectional study compares financial measures, including margins and commercial prices, between critical access hospitals and other acute care hospitals and examines how these comparisons vary by system affiliation.

## Introduction

Critical access hospitals (CAHs), which have no more than 25 beds and are geographically separate from neighboring hospitals, have both unique financing models and financial pressures that have contributed to closures or consolidation with health systems.^1,2,3^ The association between CAH financial status and system affiliation, and how CAHs compare to non-CAHs, is not well understood. System affiliation may impact negotiation leverage with commercial insurers and, thus, improve financial margins. In this cross-sectional study, we compare hospital financial measures, including margins and commercial prices, between CAHs and non-CAHs and examine how these comparisons vary by system affiliation.

## Methods

Data on hospital overall operating margins and payer-specific operating margins (Medicare, Medicaid, and commercial insurance) were obtained from the National Academy for State Health Policy (NASHP) Hospital Cost Tool, which reports financial metrics for approximately 4500 US hospitals over the 2016 to 2022 period.^4^ NASHP data incorporate data from hospital cost report information collected by the Centers for Medicare & Medicaid Services (CMS). System affiliation was identified using the Agency for Healthcare Research and Quality Compendium. Data on hospital prices were obtained from a collection of medical claims data obtained from employer-sponsored insurance plans and state all-payer claims databases.^5^ These data include price information, measured using claim-allowed amounts, from approximately 4100 US hospitals over the pooled 2020 to 2022 period. Separately for inpatient and outpatient services, prices were standardized using CMS-reported service weights. Both sets of data are publicly available. In both datasets, hospitals were limited to general medical and surgical hospitals, including both CAHs and non-CAHs, and excluded specialty hospitals. Multivariate linear regressions were used to compare financial outcomes by hospital type, with adjustments for number of beds, Medicaid, Medicare, and commercial insurer payer mix. This study followed STROBE reporting guidelines.

## Results

The number of hospitals included in the study decreased from 3571 in 2011 to 3488 in 2022. In 2022, 507 (14.5%) hospitals were independent CAHs, 590 (16.9%) were system-affiliated CAHs, 260 (7.5%) were independent non-CAHs, and 2131 (61.1%) were system-affiliated non-CAHs. The share of CAHs that were system affiliated increased from 466 (49.6%) in 2011 to 590 (53.8%) in 2022.

**Figure 1.**
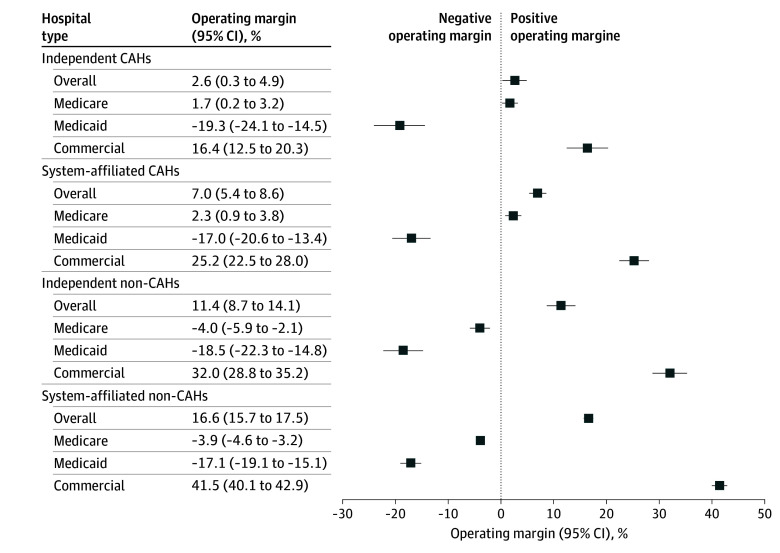
Regression-Adjusted Overall and Payer-Specific Operating Margins by Hospital Type This figure presents regression-adjusted overall operating and payer-specific margins over the 2016 to 2022 period between independent critical access hospitals (CAHs), system-affiliated CAHs, independent non-CAHs, and system-affiliated CAHs. This figure uses data from the National Academy for State Health Policy Hospital Cost Tool, which aggregates data from Centers for Medicare & Medicaid Services hospital cost reports. Hospitals with 100% positive or negative operating margins (overall and payer specific) were excluded. The National Academy for State Health Policy data links to the Agency for Healthcare Research and Quality Compendium, which was used to identify hospital system affiliation. Predicted values are shown from a multivariate linear regression that includes controls for geographic market (state)-by-year fixed effect interactions, number of beds, and payer mix, with standard errors clustered at the state level.

**Figure 2.**
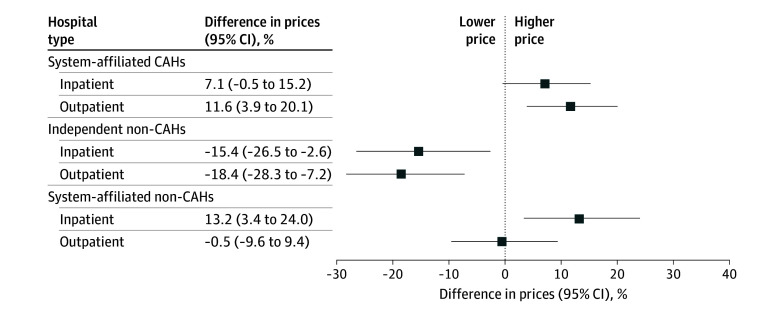
Regression-Adjusted Differences in Standardized Inpatient and Outpatient Commercial Prices Relative to Independent Critical Access Hospitals (CAHs) This figure presents regression-adjusted differences in standardized inpatient and outpatient commercial prices over the pooled 2020 to 2022 period. Price differences, relative to independent CAHs, are presented for system-affiliated CAHs, independent non-CAHs, and system-affiliated CAHs. Price differences are compared using a linear regression that includes controls for geographic market (state) fixed-effect controls, number of beds, and payer mix, with standard errors clustered at the state level. Both inpatient and outpatient standardized price–dependent variables were log transformed and converted into percent differences by taking the exponent of the regression coefficients.

Independent CAHs averaged 2.6% (95% CI, 0.3%-4.9%) overall operating margins, compared to 7.0% (95% CI, 5.4%-8.6%) for system-affiliated CAHs, 11.4% (95% CI, 8.7%-14.1%) for independent non-CAHs, and 16.6% (95% CI, 15.7%-17.5%) for system-affiliated non-CAHs (Figure 1). Medicare operating margins were positive (approximately 2%) for CAHs but negative (approximately 4%) for non-CAHs. Medicaid operating margins were similar at approximately −18% for all hospitals. Commercial operating margins were 16.4% (95% CI, 12.5%-20.3%) for independent CAHs, 25.2% (95% CI, 22.5%-28.0%) for system-affiliated CAHs, 32.0% (95% CI, 28.8%-35.2%) for independent non-CAHs, and 41.5% (95% CI, 40.4%-43.0%) for system-affiliated non-CAHs (Figure 2). Relative to independent CAHs, standardized inpatient commercial prices were 7.1% higher (95% CI, −0.5% to 15.2%) among system-affiliated CAHs, 15.4% lower (95% CI, −26.5% to −2.6%) among independent non-CAHs, and 13.2% higher (95% CI, 3.4%-24.0%) among system-affiliated non-CAHs; for outpatient services, the relative differences were 11.7% (95% CI, 3.9%-20.1%), −18.4% (95% CI, −28.3% to −7.2%), and −0.5% (95% CI, −9.6% to 9.4%), respectively.

## Discussion

CAHs have unique financial pressures, particularly in the years following the COVID-19 pandemic.^6^ In this cross-sectional study, CAHs had lower overall operating margins but higher Medicare operating margins than other acute care hospitals. Operating margins for system-affiliated CAHs were 63% higher than for independent CAHs (4.4–percentage point absolute difference). Relative to independent hospitals, system-affiliated hospitals had higher commercial prices. Limitations of this study include use of financial metrics derived from CMS cost report data, rather than audited financial statements, and the lack of national hospital price data.

These results suggest that when comparing hospital financial performance, considering system affiliation is critical. Particularly in commercial markets, system affiliation enables aggregations of negotiation leverage, leading to higher prices. Policymakers interested in improving patient access to hospital care in rural areas should consider the impact of consolidation for both CAHs and non-CAHs, such as whether the increased commercial prices associated with system affiliation correspondingly lead to improved and sustained patient access to care.

## Notes

Supplement. Data Sharing Statement
