# Supplementary material for: Financial Performance Gaps Between Critical Access Hospitals and Other Acute Care Hospitals
Source: JAMA Health Forum. 2024 Dec 20;5(12):e243959. doi: 10.1001/jamahealthforum.2024.3959 (PMC11662246; doi:10.1001/jamahealthforum.2024.3959)
Supplement: Supplement. — Data Sharing Statement [file jamahealthforum-e243959-s001.pdf]

Data Sharing Statement

Whaley. Financial Performance Gaps Between Critical Access Hospitals and Other Acute Care Hospitals. *JAMA Health Forum*. Published December 20, 2024.
doi:10.1001/jamahealthforum.2024.3959

Data
Data available: Yes
Data types: Data (not involving human participants)
How to access data: Data from CMS cost reports will be made available by request.
When available: With publication

Supporting Documents
Document types: Statistical/analytic code
How to access documents: Statistical/analytic code analyzing CMS cost report data will be made available by request.
When available: With publication

Additional Information
Who can access the data: Data/code will be made available by request.
Types of analyses: Data/code will be made available by request.
Mechanisms of data availability: Data/code will be made available by request.
